# Increased nuchal translucency thickness and normal chromosomal microarray: Danish nationwide cohort study

**DOI:** 10.1002/uog.29198

**Published:** 2025-02-27

**Authors:** K. Gadsbøll, N. Brix, P. Sandager, O. B. Petersen, A. P. Souka, K. H. Nicolaides, I. Vogel

**Affiliations:** ^1^ Center for Fetal Medicine, Pregnancy and Ultrasound, Department of Gynecology, Fertility and Obstetrics Copenhagen University Hospital Rigshospitalet, Copenhagen Denmark; ^2^ Department of Clinical Genetics Aarhus University Hospital Aarhus Denmark; ^3^ Department of Public Health, Research Unit for Epidemiology Aarhus University Aarhus Denmark; ^4^ Department of Obstetrics and Gynecology, Center for Fetal Medicine Aarhus University Hospital Aarhus Denmark; ^5^ Center for Fetal Diagnostics Aarhus University Aarhus Denmark; ^6^ Department of Clinical Medicine Aarhus University Aarhus Denmark; ^7^ Faculty of Health and Medical Sciences, Department of Clinical Medicine University of Copenhagen Copenhagen Denmark; ^8^ Department of Obstetrics and Gynecology, Alexandra Hospital National and Kapodistrian University of Athens Athens Greece; ^9^ Harris Birthright Research Centre for Fetal Medicine, King's College Hospital London UK

**Keywords:** chromosomal aberration, chromosomal microarray, combined first‐trimester screening, nuchal translucency, prenatal screening, risk assessment

## Abstract

**Objective:**

To assess the outcome of pregnancies with increased fetal nuchal translucency (NT) thickness and a normal result from chromosomal microarray (CMA) *vs* conventional karyotyping.

**Methods:**

This was a Danish nationwide registry‐based cohort study of all singleton pregnancies seen for combined first‐trimester screening between 2008 and 2018. Data on NT thickness and pregnancy outcome were retrieved from the Danish Fetal Medicine Database, whereas data on cytogenetic and molecular karyotypes were retrieved from the Danish Cytogenetic Central Register. Pregnancies were stratified according to NT thickness, and we computed the prevalence of chromosomal aberration, termination of pregnancy (due to non‐genetic abnormal findings aside from increased NT), pregnancy loss, major congenital malformation and unaffected live birth (live birth ≥ 24 weeks' gestation with no chromosomal aberration or major congenital malformation diagnosed). The prevalence of the different outcomes was further estimated for pregnancies with increased NT (≥ 3.5 mm) and a normal CMA result. Finally, to assess the impact of CMA compared with conventional karyotyping for increased NT, we compared the prevalence of chromosomal aberrations and each pregnancy outcome between the periods 2008–2012 and 2014–2018 (during which < 3% and > 60%, respectively, of pregnancies with increased NT were examined using CMA).

**Results:**

We identified 557 896 pregnancies with a NT measurement for which outcome data were registered. Fetal NT was ≥ 3.5 mm in 3717 (0.7%) pregnancies, of which 3368 (91%) underwent genetic examination. The prevalence of chromosomal aberrations increased significantly with increasing NT thickness, from 21% in pregnancies with NT of 3.5–4.4 mm to 69% in pregnancies with NT ≥ 6.5 mm. Trisomies 21, 18 and 13 accounted for the majority of chromosomal aberrations diagnosed in all subgroups of increased NT (range, 61–87%). In pregnancies with increased NT and a normal CMA result, the prevalence of unaffected live birth decreased significantly from 87% for NT of 3.5–4.4 mm to 29% for NT ≥ 6.5 mm. Increased uptake of CMA during 2014–2018 compared with 2008–2012 slightly increased the detection of submicroscopic aberrations. However, a normal CMA result, compared with a normal result from conventional karyotyping, did not substantially improve the prognosis in pregnancies with increased NT.

**Conclusions:**

Our study reaffirms the association between increased NT and chromosomal aberrations. Although CMA improves diagnostic resolution in pregnancies with increased NT, a normal test result does not substantially impact the prevalence of unaffected live births. This highlights the ongoing need for accurate clinical guidance and continued research, especially as whole‐genome sequencing is increasingly adopted in prenatal care. © 2025 The Author(s). *Ultrasound in Obstetrics & Gynecology* published by John Wiley & Sons Ltd on behalf of International Society of Ultrasound in Obstetrics and Gynecology.

## INTRODUCTION

Subcutaneous accumulation of fluid in the fetal neck is a normal phenomenon that occurs during the first trimester of pregnancy, and is visualized by ultrasonography as the nuchal translucency (NT). Increased NT thickness is associated strongly with chromosomal aneuploidies^1^, ^2^, ^3^, ^4^. Several factors contribute to increased NT, including anomalies in the fetal circulatory^5^, ^6^, ^7^, ^8^ and lymphatic^9^, ^10^, ^11^, ^12^, ^13^ systems and alterations in the extracellular matrix^14^, ^15^. Even in the presence of a normal karyotype, fetuses with increased NT are at increased risk of several structural malformations, genetic syndromes and adverse pregnancy outcomes^7^, ^16^, ^17^, ^18^. In 2005, Souka *et al*. reported that the prevalence of chromosomal defects diagnosed by conventional karyotyping increased exponentially from 21% in fetuses with NT between 3.5 and 4.4 mm to 65% in fetuses with NT ≥ 6.5 mm^16^. The risk of fetal death and major structural malformation increased similarly. Hence, the probability of delivering a liveborn child with a normal karyotype and no major congenital malformations decreased from 70% to 15% for fetuses with NT between 3.5 mm to 4.4 mm and NT ≥ 6.5 mm, respectively.

Since the introduction of chromosomal microarray (CMA) in the prenatal setting, increased NT has been associated with several submicroscopic copy‐number variants (CNVs) not detectable by conventional karyotyping^19^, ^20^, ^21^, ^22^. In pregnancies with increased NT, CMA likely identifies more cases with a fetal chromosomal aberration that might affect the pregnancy outcome compared to conventional karyotyping. We hypothesized that the overall prognosis in pregnancies with a normal CMA result may now be better than that reported previously in pregnancies with increased NT and a normal result from conventional karyotyping.

To improve prenatal counseling, we aimed to assess the outcomes of pregnancies with increased NT and a normal CMA result. Furthermore, we evaluated the impact of CMA compared with conventional karyotyping on diagnostic resolution for pregnancies with increased NT and, finally, we estimated the prevalence of various subtypes of chromosomal aberrations.

## METHODS

### Data source

The Danish Fetal Medicine Database (DFMD) stores data on all pregnancies seen for prenatal screening in Denmark since 2008^23^, ^24^. Data on maternal characteristics, markers for combined first‐trimester screening (cFTS), screening results, fetal biometry and suspected prenatal malformations are uploaded automatically from local fetal medicine departments (Astraia GmbH, Munich, Germany) to the DFMD. The DFMD is further supplied with data on pregnancy complications, pregnancy outcome, delivery details and neonatal characteristics from the Danish Medical Birth Registry^25^, and with data on postnatally detected malformations from the Danish National Patient Registry^26^.

Data on all cytogenetic and molecular karyotypes analyzed at all Danish Departments of Clinical Genetics are recorded in the Danish Cytogenetic Central Register (DCCR) by the DCCR study group^27^, ^28^. Karyotypes obtained from prenatal invasive sampling, from fetal tissue following pregnancy loss or termination of pregnancy (TOP) and from postnatal samples are included. Pre‐ and postnatal genetic testing is covered financially by the Danish healthcare system on indication and, thus, the DCCR holds data on virtually all genetic analyses performed nationwide since the 1960s.

Individual‐level data are linked across the national registries using a personal identification number (the CPR number) that is assigned to all Danish citizens upon birth or immigration^29^. The use of data for this study was approved by the Danish Data Protection Agency (approval number: p‐2024‐15588). Registry‐based studies do not require approval from the Danish Research Ethics Committee system.

### Study patients and design

We conducted a nationwide registry‐based cohort study. We retrieved data from the DFMD on all singleton pregnancies that underwent cFTS in Denmark between 1 January 2008 and 31 December 2018, with a registered NT measurement. From the DCCR, we identified all cytogenetic and molecular analyses performed on prenatal samples (chorionic villus sampling or amniocentesis), postnatal samples or fetal tissue following pregnancy loss or termination. To ensure consistent postnatal follow‐up, only karyotypes obtained before the age of 2 years were included. An updated version of a previously published algorithm^30^ was used to bulk‐sort each karyotype into one of six aberration categories: triploidy; common trisomy (trisomies 21, 18 and 13); monosomy X; other sex‐chromosome aneuploidy (e.g. XXX, XXY, XYY); CNVs (i.e. microdeletions and microduplications, unbalanced translocations and marker chromosomes); and pooled rare autosomal trisomies and mosaicisms. Postnatally detected structural malformations were classified according to the European Platform on Rare Disease Registration (EUROCAT)^31^, and minor malformations were excluded^32^. Pregnancy loss was defined as fetal demise at any point between registration at cFTS and delivery. Unaffected live birth was defined as live birth ≥ 24 + 0 weeks' gestation with no chromosomal aberration or major congenital malformation diagnosed.

### Prenatal screening in Denmark

Since 2004, Danish pregnant women have been offered cFTS between 11 + 2 and 13 + 6 weeks' gestation, accompanied by a second‐trimester ultrasound scan for anomalies at 18 + 0 to 21 + 6 weeks^33^, ^34^. Prenatal screening is freely accessible to all, covered by the tax‐financed public healthcare system, with uptake exceeding 90% for cFTS and 95% for the second‐trimester anomaly scan^24^, ^35^. All screening is carried out by sonographers certified by the Fetal Medicine Foundation to perform the NT scan.

Women with a family history of chromosomal aberration or a previous affected pregnancy are offered early invasive diagnostic testing, often before the first‐trimester scan. Following cFTS, invasive diagnostic testing is offered in the case of a high‐risk screening result (trisomy 21 risk > 1 in 300 or trisomy 18/13 risk > 1 in 150). Women with low‐risk pregnancies with increased fetal NT (≥ 3.5 mm) are also offered invasive diagnostic testing and an additional early scan for structural malformations at 16–18 weeks' gestation. Following the second‐trimester routine anomaly scan at 18–21 weeks' gestation, invasive diagnostic testing is offered in the case of a major structural malformation or severe growth restriction. Since 2013, non‐invasive prenatal testing for trisomies 21, 18 and 13 and monosomy X has been offered as an alternative to invasive testing for women with a high‐risk cFTS result and NT < 3.5 mm. However, the uptake of non‐invasive prenatal testing in Denmark is low compared with that in other countries^36^.

In Denmark, CMA was introduced into the prenatal diagnostic setting in 2011 and was adopted for various indications across the Danish regions^37^. In 2013, the Danish Fetal Medicine Society recommended CMA over conventional karyotyping for all pregnancies with NT ≥ 3.5 mm, structural malformations or small fetal biometry. Since 2018, CMA has been recommended for all prenatal indications^38^ but was not implemented fully in all Danish hospitals until September 2020. However, in some cases, quantitative fluorescence polymerase chain reaction (QF‐PCR) or conventional karyotyping may still be used as the initial approach, focusing on detecting the common trisomies, with CMA reserved for cases with a normal initial result. Since its introduction, CMA has been used with a resolution of approximately 50 kb (180K comparative genomic hybridization (CGH) array is used most frequently). Early in the implementation process, QF‐PCR or multiplex ligation‐dependent probe amplification may have been used as the first‐line method before CMA, but this has been discontinued due to low detection yield and high cost.

In Denmark, TOP is a legal right for all women until 11 + 6 weeks' gestation. Beyond 12 + 0 weeks' gestation, the legality of TOP is evaluated on a case‐by‐case basis by a regional abortion council. In pregnancies with NT ≤ 6 mm, the council generally requires the presence of additional anomalies, such as chromosomal aberrations or structural malformations, to authorize TOP. Hence, in chromosomally normal pregnancies with NT of 3.5–6 mm resulting in TOP, fetal anomalies other than increased NT can generally be assumed to be present.

### Statistical analysis

The study cohort was stratified into five subgroups according to NT thickness: < 3.5 mm, 3.5–4.4 mm, 4.5–5.4 mm, 5.5–6.4 mm and ≥ 6.5 mm. Pregnancy outcome was assigned to one of five categories in a hierarchical order: (1) chromosomal aberration; (2) TOP; (3) pregnancy loss; (4) live birth without chromosomal aberration but with major congenital malformation; and (5) unaffected live birth (birth ≥ 24 + 0 weeks' gestation without diagnosed chromosomal aberration or major congenital malformation). If a pregnancy had more than one outcome (e.g. a chromosomal aberration followed by TOP), the pregnancy was categorized under the highest‐ranking outcome (in this case, chromosomal aberration) and did not contribute to the lower‐ranking category (TOP). As a result, pregnancies categorized as TOP were terminated because of abnormal findings other than a chromosomal aberration.

To address the increasing use of prenatal CMA, the study period was divided into two periods (2008–2012 and 2014–2018), and the prevalence of each outcome was estimated within each NT subgroup. During these two periods, < 3% and > 60% of pregnancies with increased NT were examined using CMA, respectively (Figure S1). The distribution of pregnancy outcomes was also computed for pregnancies with increased NT and a normal CMA result.

Exact binomial 95% CIs were computed using the Clopper–Pearson interval for categorical variables. Groups were compared using Fisher's exact test. *P*‐values < 0.05 were considered statistically significant. Data management, analysis and figure production were performed using R statistical software version 4.2.2^39^.

## RESULTS

We retrieved data on 613 278 pregnancies, of which 563 136 (91.8%) had a NT measurement registered at a crown–rump length of 45–84 mm. We excluded 5240 (0.9%) pregnancies due to missing outcome data, so the final study cohort comprised 557 896 pregnancies. Fetal NT was ≥ 3.5 mm in 3717 (0.7%) pregnancies, of which 3368 (90.6%) underwent genetic examination. Before 2013, few pregnancies with increased NT underwent CMA analysis of an invasive sample or fetal tissue. The proportion of pregnancies for which CMA was performed increased during the study period, from 19% in 2013 to 77% in 2018, in accordance with national recommendations^38^ (Figure S1).

**Figure 1:**
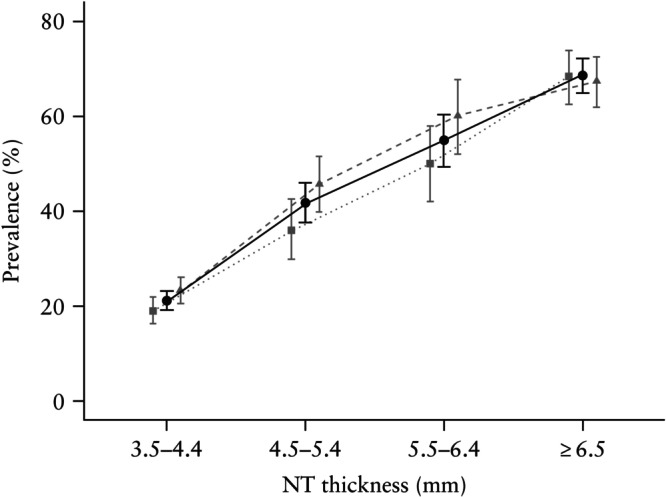
Prevalence, with 95% CI, of any pathogenic chromosomal aberration diagnosed prenatally or from fetal tissue following pregnancy loss or termination, stratified by nuchal translucency (NT) thickness, for the periods 2008–2018 (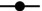), 2008–2012 (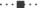) and 2014–2018 (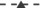).

Table 1 displays the maternal and pregnancy characteristics for each NT thickness subgroup. In the entire cohort, 3916 (0.70% (95% CI, 0.68–0.72%)) pregnancies were diagnosed with a chromosomal aberration at any point. Among genetically examined pregnancies (prenatally or fetal tissue) with increased NT, the overall prevalence of chromosomal aberrations was 37.2% (95% CI, 35.5–38.8%). The prevalence of chromosomal aberrations increased significantly with increasing NT thickness, from 21.1% in pregnancies with NT of 3.5–4.4 mm to 68.6% in pregnancies with NT ≥ 6.5 mm (Figure 1). The prevalence of chromosomal aberrations in the subgroups with NT of 3.5–4.4 mm, 4.5–5.4 mm and 5.5–6.4 mm was significantly higher in the period 2014–2018 compared with the period 2008–2012.

**Table 1:** Maternal and pregnancy characteristics in entire cohort and in each subgroup of increased first‐trimester nuchal translucency (NT) thickness

		NT ≥ 3.5 mm (*n* = 3717)			
Characteristic	All pregnancies (*n* = 557 896)	3.5–4.4 mm (*n* = 1977)	4.5–5.4 mm (*n* = 612)	5.5–6.4 mm (*n* = 359)	≥ 6.5 mm (*n* = 769)
Maternal age (years)	30 (26–33)	31 (27–35)	32 (28–36)	32 (28–37)	32 (28–37)
BMI (kg/m^2^)*	23 (20–26)	23 (21–26)	23 (20–26)	23 (21–26)	23 (20–25)
Caucasian ethnicity	515 824/550 119 (93.8)	1824/1941 (94.0)	569/599 (95.0)	336/350 (96.0)	702/753 (93.2)
Current smoker	50 532/556 484 (9.1)	217/1968 (11.0)	63/606 (10.4)	34/357 (9.5)	48/757 (6.3)
Nulliparous	193 947/433 575 (44.7)	681/1625 (41.9)	187/495 (37.8)	114/284 (40.1)	247/617 (40.0)
Spontaneous conception	510 313/551 034 (92.6)	1779/1954 (91.0)	559/603 (92.7)	321/354 (90.7)	678/747 (90.8)
Genetic testing performed	34 728 (6.2)	1814 (91.8)	572 (93.5)	327 (91.1)	655 (85.2)
Prenatally†	29 239 (5.2)	1775 (89.8)	533 (87.1)	282 (78.6)	469 (61.0)
After pregnancy loss orTOP (fetal tissue)	2428 (0.4)	24 (1.2)	33 (5.4)	44 (12.3)	184 (23.9)
Postnatally‡	3061 (0.5)	15 (0.8)	6 (1.0)	< 3 (0.3)	< 3 (0.3)

Data are given as median (interquartile range), *n*/*N* (%) or *n* (%).

* Data for body mass index (BMI) missing for 12 031 of all pregnancies, 63 of those with NT of 3.5–4.4 mm, 26 of those with NT of 4.5–5.4 mm, 19 of those with NT of 5.5–6.4 mm and 35 of those with NT ≥ 6.5 mm.

† Chorionic villus sampling or amniocentesis.

‡ Before 2 years of age.

TOP, termination of pregnancy.

### Subtypes of chromosomal aberration

The types of chromosomal aberration diagnosed within each subgroup of increased NT are displayed in Table S1. Comparisons between the two time periods (2008–2012 *vs* 2014–2018) are also presented. Trisomies 21, 18 and 13 accounted for the majority of chromosomal aberrations diagnosed in all subgroups (range, 60.7–86.6%). In pregnancies with NT ≥ 6.5 mm, monosomy X comprised 29.7% of aberrations, compared with < 6% in all subgroups with NT < 6.5 mm. Pathogenic CNVs comprised a significantly larger proportion of the aberrations diagnosed in 2014–2018 compared with 2008–2012 (9.8% *vs* 4.1%, *P* < 0.001). In the period 2014–2018, the contribution of pathogenic CNVs decreased with increasing NT, from 15.5% in pregnancies with NT of 3.5–4.4 mm to 4.2% in pregnancies with NT of 5.5–6.4 mm. Conventional cell‐free fetal DNA testing (assuming 100% sensitivity for the common trisomies and monosomy X) would miss 22%, 18%, 7% and 12% of the chromosomal aberrations diagnosed during the period 2014–2018 in pregnancies with NT of 3.5–4.4 mm, 4.5–5.4 mm, 5.5–6.4 mm and ≥ 6.5 mm, respectively.

### Chromosomal microarray for increased nuchal translucency

In pregnancies with increased NT thickness and a normal CMA result (excluding postnatal CMA) (*n* = 966), the prevalence of unaffected live birth decreased significantly from 87.2% for NT of 3.5–4.4 mm to 29.0% for NT ≥ 6.5 mm (Table 2). This decrease was driven mainly by an increase in the prevalence of TOP (generally due to an additional abnormal finding in the case of NT ≤ 6 mm), which increased significantly with increasing NT thickness, from 4.6% for NT of 3.5–4.4 mm to 52.7% for NT ≥ 6.5 mm. The prevalence of pregnancy loss increased from 1.8% in those with NT of 3.5–4.4 mm to 11.8% in those with NT ≥ 6.5 mm. The prevalence of major congenital malformation was slightly increased in the subgroups with NT of 4.5–5.4 mm and 5.5–6.4 mm. In pregnancies with NT of 3.5–6.0 mm, for which TOP was generally indicated due to fetal malformations other than the increased NT, the prevalence of unaffected live birth in pregnancies reaching 14, 18, 24 and 28 weeks' gestation was, respectively, 85.6% (ranging from 65.0% to 87.8% in the three NT subgroups), 89.7% (ranging from 74.3% to 90.9% in the three NT subgroups), 91.7% (ranging from 81.3% to 92.6% in the three NT subgroups) and 92.1% (ranging from 81.3% to 93.0% in the three NT subgroups) (Table 3).

**Table 2:** Pregnancy outcomes following a normal chromosomal microarray result, in all pregnancies and in those with increased first‐ trimester nuchal translucency (NT) thickness

				Liveborn	
Group	*n*	TOP due to additional fetal anomaly	Pregnancy loss	Major congenital malformation	Unaffected
NT ≥ 3.5 mm	966	117 (12.1 (10.1–14.3))	34 (3.5 (2.5–4.9))	66 (6.8 (5.3–8.6))	749 (77.5 (74.8–80.1))
3.5–4.4 mm	650	30 (4.6 (3.1–6.5))	12 (1.8 (1.0–3.2))	41 (6.3 (4.6–8.5))	567 (87.2 (84.4–89.7))
4.5–5.4 mm	158	20 (12.7 (7.9–18.9))	8 (5.1 (2.2–9.7))	12 (7.6 (4.0–12.9))	118 (74.7 (67.2–81.3))
5.5–6.5 mm	65	18 (27.7 (17.3–40.2))	3 (4.6 (1.0–12.9))	7 (10.8 (4.4–20.9))	37 (56.9 (44.0–69.2))
≥ 6.5 mm	93	49 (52.7 (42.1–63.1))	11 (11.8 (6.0–20.2))	6 (6.5 (2.4–13.5))	27 (29.0 (20.1–39.4))
All pregnancies	557 896	4706 (0.8 (0.8–0.9))	5220 (0.9 (0.9–1.0))	17 119 (3.1 (3.0–3.1))	530 851 (95.2 (95.1–95.2))

Data are given as *n* or *n* (% (95% CI)). Fetal malformation generally had to be present for termination of pregnancy (TOP) to be permitted in a genetically normal pregnancy with NT of 3.5–6 mm; however, TOP was permitted for pregnancies with isolated first‐trimester NT > 6 mm, so additional anomalies were not necessarily present in this group.

**Table 3:** Outcomes of pregnancies with a first‐trimester nuchal translucency (NT) thickness of 3.5–6.0 mm and a normal chromosomal microarray result, stratified according to pregnancy reaching 14, 18, 24 and 28 weeks' gestation

Group	*n*	Pregnancy loss	Fetal anomaly*	Unaffected live birth
*≥ 14 weeks' gestation*				
NT 3.5–6.0 mm	831	17 (2.0 (1.2–3.3))	103 (12.4 (10.2–14.8))	711 (85.6 (83.0–87.9))
3.5–4.4 mm	646	12 (1.9 (1.0–3.2))	67 (10.4 (8.13–13.0))	567 (87.8 (85.0–90.2))
4.5–5.4 mm	145	4 (2.8 (0.76–6.9))	23 (15.9 (10.3–22.8))	118 (81.4 (74.1–87.4))
5.5–6.0 mm	40	< 3 (2.5 (0.1–13.2))	13 (32.5 (18.6–49.1))	26 (65.0 (48.3–79.4))
*≥ 18 weeks' gestation*				
NT 3.5–6.0 mm	793	13 (1.6 (0.9–2.8))	69 (8.7 (6.8–10.9))	711 (89.7 (87.3–91.7))
3.5–4.4 mm	624	10 (1.6 (0.8–2.9))	47 (7.5 (5.6–9.9))	567 (90.9 (88.3–93.0))
4.5–5.4 mm	134	< 3 (1.5 (0.2–5.3))	14 (10.4 (5.83–16.9))	118 (88.1 (81.3–93.0))
5.5–6.0 mm	35	< 3 (2.9 (0.1–14.9))	8 (22.9 (10.4–40.1))	26 (74.3 (56.7–87.5))
*≥ 24 weeks' gestation*				
NT 3.5–6.0 mm	775	5 (0.6 (0.2–1.5))	59 (7.6 (5.9–9.7))	711 (91.7 (89.6–93.6))
3.5–4.4 mm	612	4 (0.7 (0.2–1.7))	41 (6.7 (4.9–9.0))	567 (92.6 (90.3–94.6))
4.5–5.4 mm	131	< 3 (0.8 (0.0–4.18))	12 (9.2 (4.8–15.5))	118 (90.1 (83.6–94.6))
5.5–6.0 mm	32	< 3 (0.0 (0.0–10.9))	6 (18.8 (7.2–36.4))	26 (81.3 (63.6–92.8))
*≥ 28 weeks' gestation*				
NT 3.5–6.0 mm	772	< 3 (0.3 (0.0–0.9))	59 (7.6 (5.9–9.8))	711 (92.1 (90.0–93.9))
3.5–4.4 mm	610	< 3 (0.3 (0.0–1.2))	41 (6.7 (4.9–9.0))	567 (93.0 (90.6–94.9))
4.5–5.4 mm	130	< 3 (0.0 (0.0–2.8))	12 (9.2 (4.9–15.6))	118 (90.8 (84.4–95.1))
5.5–6.0 mm	32	< 3 (0.0 (0.0–10.9))	6 (18.8 (7.2–36.4))	26 (81.3 (63.6–92.8))

Data are given as *n* or *n* (% (95% CI)).

* Includes termination of pregnancy and live birth with major congenital malformation.

Fetal malformation generally had to be present for termination of pregnancy to be permitted in a genetically normal pregnancy with NT of 3.5–6.0 mm.

**Figure 2:**
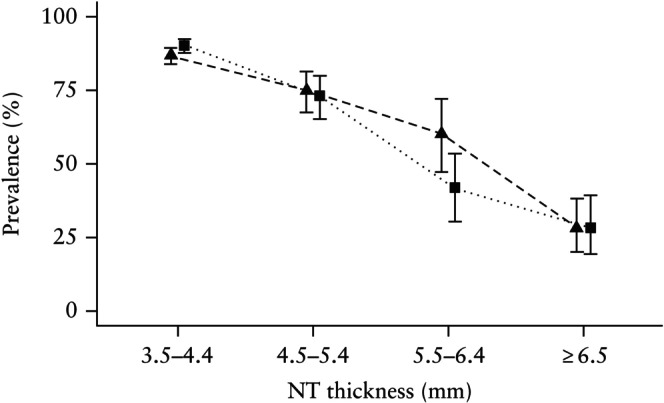
Prevalence, with 95% CI, of unaffected live birth in pregnancies with increased nuchal translucency (NT) thickness and a normal result from genetic testing, in the periods 2008–2012 (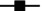) and 2014–2018 (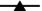).

Table 4 shows comparison of pregnancy outcomes across NT subgroups for the periods 2008–2012 (< 3% tested by CMA) and 2014–2018 (> 60% tested by CMA). The prevalence of chromosomal aberrations in pregnancies with increased NT was significantly higher in the second period (39.1% *vs* 34.5%, *P* = 0.01), resulting in a slightly lower prevalence of unaffected live births (47.1% *vs* 51.0%, *P* = 0.04). In pregnancies with increased NT without a chromosomal aberration, the overall prevalence of unaffected live birth was similar in the two periods (783/1012 (77.4%) in 2014–2018 *vs* 722/927 (77.9%) in 2008–2012, *P* = 0.83). However, in genetically normal pregnancies with NT of 5.5–6.4 mm, a significantly higher prevalence of unaffected live birth was observed in the later period (38/63 (60.3%) *vs* 31/75 (41.3%), *P* = 0.04) (Figure 2).

**Table 4:** Prevalence of chromosomal aberration, termination of pregnancy (TOP), pregnancy loss, fetal major congenital malformation and unaffected live birth in all pregnancies and in genetically examined pregnancies with increased first‐trimester nuchal translucency (NT) thickness during 2008–2012 and 2014–2018

Group	*n*	Chromosomal aberration diagnosed at any point*	TOP due to additional fetal anomaly†	Pregnancy loss	Live birth with major congenital malformation	Unaffected live birth
*2008–2012*						
NT ≥ 3.5 mm	1416	489 (34.5 (32.1–37.1))	132 (9.3 (7.9–11.0))	29 (2.0 (1.4–2.9))	44 (3.1 (2.3–4.2))	722 (51.0 (48.3–53.6))
3.5–4.4 mm	764	146 (19.1 (16.4–22.1))	25 (3.3 (2.1–4.8))	11 (1.4 (0.7–2.6))	24 (3.1 (2.0–4.6))	558 (73.0 (69.7–76.2))
4.5–5.4 mm	234	85 (36.3 (30.2–42.8))	24 (10.3 (6.7–14.9))	7 (3.0 (1.2–6.1))	9 (3.8 (1.8–7.2))	109 (46.6 (40.1–53.2))
5.5–6.4 mm	149	74 (49.7 (41.4–58.0))	34 (22.8 (16.3–30.4))	5 (3.4 (1.1–7.7))	5 (3.4 (1.1–7.7))	31 (20.8 (14.6–28.2))
≥ 6.5 mm	269	184 (68.4 (62.5–73.9))	49 (18.2 (13.8–23.4))	6 (2.2 (0.8–4.8))	6 (2.2 (0.8–4.8))	24 (8.9 (5.8–13.0))
All pregnancies	238 681	1529 (0.64 (0.61–0.67))	982 (0.41 (0.39–0.44))	2194 (0.91 (0.88–0.96))	6480 (2.71 (2.65–2.78))	227 496 (95.3 (95.2–95.4))
*2014–2018*						
NT ≥ 3.5 mm	1661	649 (39.1 (36.7–41.5))	121 (7.3 (6.1–8.6))	37 (2.2 (1.6–3.1))	71 (4.3 (3.4–5.4))	783 (47.1 (44.7–49.6))
3.5–4.4 mm	896	210 (23.4 (20.7–26.4))	31 (3.5 (2.4–4.9))	15 (1.7 (0.9–2.8))	44 (4.9 (3.6–6.5))	596 (66.5 (63.3–69.6))
4.5–5.4 mm	292	132 (45.2 (39.4–51.1))	21 (7.2 (4.5–10.8))	6 (2.1 (0.8–4.4))	13 (4.5 (2.4–7.5))	120 (41.1 (35.4–47.0))
5.5–6.4 mm	158	95 (60.1 (52.0–67.8))	15 (9.5 (5.4–15.2))	3 (1.9 (0.4–5.5))	7 (4.4 (1.8–8.9))	38 (24.1 (17.6–31.5))
≥ 6.5 mm	315	212 (67.3 (61.8–72.5))	54 (17.1 (13.1–21.8))	13 (4.1 (2.2–7.0))	7 (2.2 (0.9–4.5))	29 (9.2 (6.3–13.0))
All pregnancies	269 429	2034 (0.75 (0.72–0.79))	1034 (0.38 (0.36–0.41))	2296 (0.85 (0.82–0.89))	8692 (3.23 (3.16–3.29))	255 373 (94.8 (94.7–94.9))

Data are given as *n* or *n* (% (95% CI)).

* Diagnosed prenatally, from fetal tissue (after pregnancy loss or TOP) or postnatally up to 2 years.

† Fetal malformation generally had to be present for TOP to be permitted in a genetically normal pregnancy with NT of 3.5–6 mm; however, TOP was permitted in pregnancies with isolated first‐trimester NT > 6 mm, so additional anomalies were not necessarily present in this group.

The overall frequency of TOP among all genetically examined pregnancies with increased NT did not change between the two periods (40.9% (579/1416) in 2008–2012 *vs* 41.3% (686/1661) in 2014–2018, *P* = 0.83). However, slightly fewer pregnancies with increased NT and a prenatally diagnosed chromosomal aberration were terminated in the later period (92.1% (386/419) in 2008–2012 *vs* 88.1% (481/546) in 2014–2018, *P* = 0.04).

## DISCUSSION

In the era of prenatal CMA, this study reassesses the implications of increased NT thickness, with a particular focus on pregnancies with a normal CMA result. Our findings echo the pivotal insights of Souka *et al*.^16^ from 2005, while providing contemporary data that should be used for prenatal counseling in the context of the widespread use of CMA.

### Principal findings and comparison with other studies

Our study reaffirms the correlation between increased NT and chromosomal abnormalities^4^, ^16^, ^17^. Although the introduction of CMA moderately increased the detection of pathogenic aberrations in pregnancies with increased NT, a normal CMA result did not substantially improve the prognosis compared with a normal result from conventional karyotyping. However, in the subgroup with NT measuring 5.5–6.4 mm without chromosomal aberrations, the prevalence of unaffected live birth was 1.5 times higher in 2014–2018 compared with 2008–2012. This increase was likely associated with the greater uptake of CMA, leading to a ‘healthier’ population with a normal genetic analysis in the later period.

From 2008 to 2012, the prevalence of chromosomal aberrations was comparable to that described by Souka *et al*.^16^. Furthermore, the proportion of common trisomies and monosomy X in our cohort mirrors the distributions reported previously^16^, reinforcing the significance of these anomalies in the context of increased NT. From 2014 to 2018, most pregnancies with increased NT in Denmark were examined prenatally with CMA, allowing for greater detection of submicroscopic aberrations. This likely contributed to the increased prevalence of chromosomal aberrations, particularly CNVs, observed during 2014–2018. In this period, pathogenic CNVs accounted for 10% of the chromosomal aberrations diagnosed in pregnancies with increased NT. This is in line with published work^40^.

### Clinical implications and future perspectives

The results from the present study may serve as a tool to estimate the residual risk in pregnancies with increased fetal NT thickness and a normal CMA result. However, caution is needed for pregnancies with NT > 6 mm, because some of the terminated pregnancies in this group were terminated due to the perceived risk associated with increased NT alone, making it difficult to predict the pregnancy outcome if not terminated.

With the ongoing implementation of prenatal whole‐genome sequencing (WGS), the clinical interpretation of increased NT is likely to be modified further. According to a recent study by Mellis *et al*.^41^, WGS demonstrates a moderate diagnostic yield for fetuses with a NT measurement above 3.5 mm. Data also indicate that fetuses with a NT measurement above 6 mm are at a significantly increased risk for RASopathies, a group of conditions marked by mutations in genes involved in the RAS/mitogen‐activated protein kinase pathway, leading, for instance, to Noonan syndrome^18^. Hence, we believe that pregnancies with increased NT will be obvious candidates for prenatal WGS in the future. The prognosis following a normal prenatal WGS result is likely to be better compared with the current standard after CMA, prompting a re‐evaluation of risk assessment, which may influence prenatal counseling in the future. The ongoing challenge for clinicians will be to integrate these advanced genomic insights into a counseling framework without having access to data on the residual risk after a normal result.

### Strengths and limitations

A major strength of our study is its nationwide registry‐based design, which encompasses over 90% of all pregnancies during the study period. Further, more than 90% of all pregnancies with increased NT underwent genetic examination. The 11‐year span of the study allowed us to compare two periods with different rates of CMA use and the corresponding impact on the prognosis of pregnancies with increased NT. A critical consideration in our study is the influence of termination based on the perceived risk associated with increased NT, as highlighted by Souka *et al*.^16^. Among pregnancies with NT ≥ 6 mm, the women who did not opt for termination likely represent a subset of pregnancies for which either the perceived risk was accepted or additional anomalies were absent, potentially skewing the overall risk assessment of increased NT. Finally, our study likely underestimates the frequency of submicroscopic chromosomal aberrations in liveborn children, as only 2 years of postnatal follow‐up were included.

### Conclusions

Although CMA improves diagnostic resolution, a normal test result does not substantially impact the incidence of unaffected live birth in pregnancies with increased NT. Our study suggests that, while CMA enhances our diagnostic ability, a normal result does not markedly improve the prognosis in pregnancies with increased NT compared to those with a normal result from conventional karyotyping. Our study confirms the foundational findings of Souka *et al*.^16^ regarding the relationship between increased NT and chromosomal abnormalities, but it also highlights the complexities and evolving nature of prenatal diagnostics and decision‐making. Our findings underscore the need for continued research and nuanced clinical guidance in managing pregnancies with increased NT.

## Supporting information


**Figure S1** Annual proportion of pregnancies with nuchal translucency thickness ≥ 3.5 mm for which genetic analysis of a prenatal sample or fetal tissue was performed using chromosomal microarray (CMA) between 2008 and 2018.

**Table S1** Categories of chromosomal aberration diagnosed prenatally or from fetal tissue in pregnancies with increased nuchal translucency thickness, overall and during the periods 2008–2012 and 2014–2018

## Data Availability

Research data are not shared due to Danish legislation.
