# Supplementary figures and images for: Increased nuchal translucency thickness and normal chromosomal microarray: Danish nationwide cohort study
Source: Ultrasound Obstet Gynecol. 2025 Feb 27;65(4):462–9. doi: 10.1002/uog.29198 (PMC11961099; doi:10.1002/uog.29198)

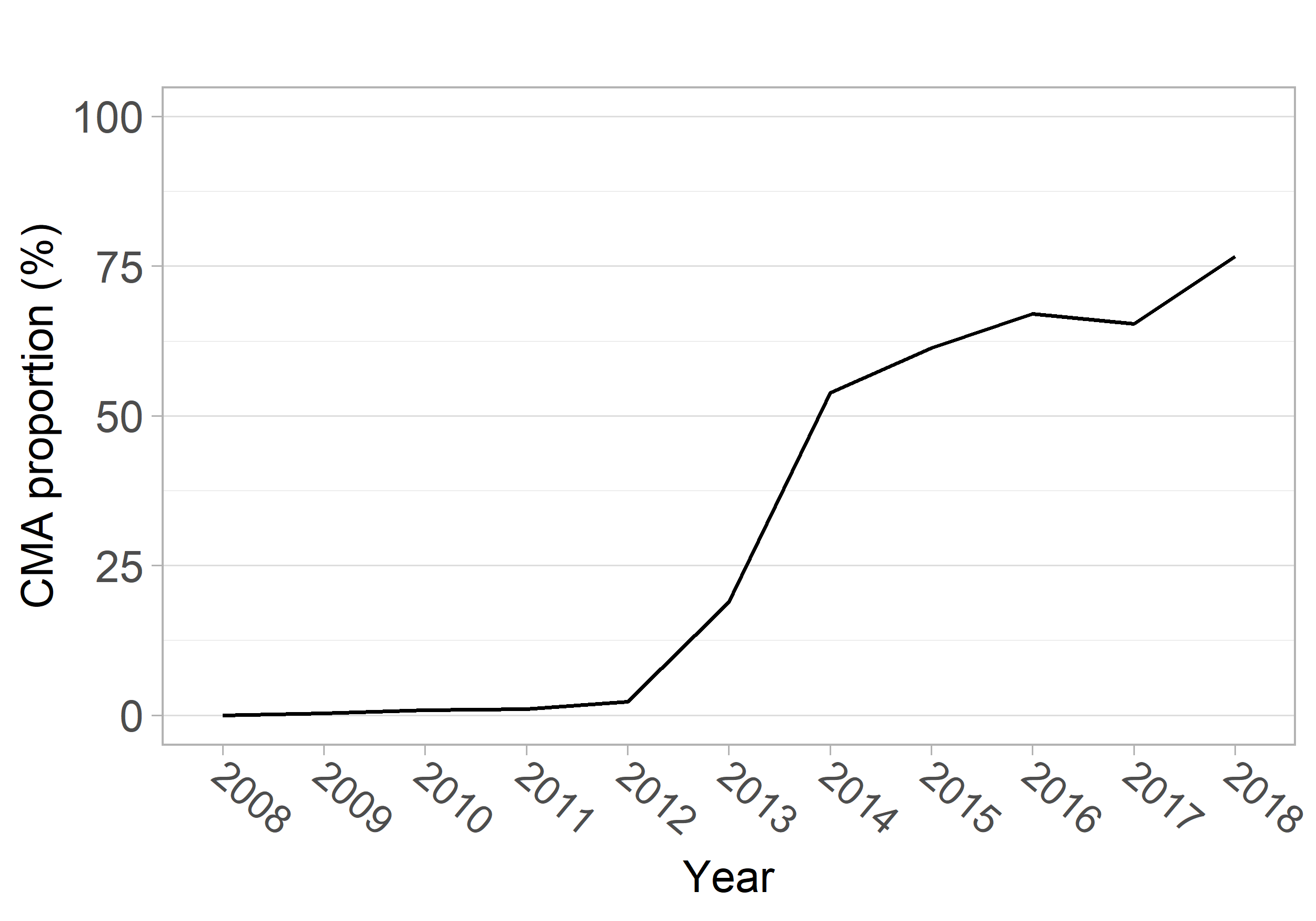

Supplement: Supplementary file 1 — Figure S1 Annual proportion of pregnancies with nuchal translucency thickness ≥ 3.5 mm for which genetic analysis of a prenatal sample or fetal tissue was performed using chromosomal microarray (CMA) between 2008 and 2018. [file UOG-65-462-s002.tiff]
