# Supplementary material for: Increased nuchal translucency thickness and normal chromosomal microarray: Danish nationwide cohort study
Source: Ultrasound Obstet Gynecol. 2025 Feb 27;65(4):462–9. doi: 10.1002/uog.29198 (PMC11961099; doi:10.1002/uog.29198)
Supplement: Supplementary file 2 — Table S1 Categories of chromosomal aberration diagnosed prenatally or from fetal tissue in pregnancies with increased nuchal translucency thickness, overall and during the periods 2008–2012 and 2014–2018 [file UOG-65-462-s001.docx]

**Table S1** Categories of chromosomal aberration diagnosed prenatally or from fetal tissue, in pregnancies with increased nuchal translucency (NT) thickness, overall and during the periods 2008–2012 and 2014–2018

| **Increased NT**  N = 1,243 | **n** | **Common trisomies** | **Triploidy** | **Monosomy X** | **Other SCA** | **CNV** | **RAT and mosaicism** |  |
| --- | --- | --- | --- | --- | --- | --- | --- | --- |
| **NT ≥3.5 mm** | 1,243 | 917  (73.8 [71.2-76.2]) | 17  (1.4 [0.8-2.2]) | 161  (13.0 [11.1-14.9]) | 15.0  (1.2 [0.7-2.0]) | 88  (7.1 [5.7-8.7]) | 45  (3.6 [2.6-4.8]) |  |
| *2008-2012* | *484* | 374  (77.3 [73.3-80.9]) | 3  (0.6 [0.1-1.8]) | 60  (12.4 [9.6-15.7]) | 7  (1.4 [0.6-3.0]) | 20  (4.1 [2.5-6.31) | 20  (4.1 [2.5-6.3]) |  |
| *2014-2018* | *645* | 461  (71.5 [67.8-74.9]) | 8  (1.2 [0.5-2.4]) | 83  (12.9 [10.4-15.7]) | 7  (1.1 [0.4-2.2]) | 63  (9.8 [7.6-12.3]) | 23  (3.6 [2.3-5.3]) |  |
| **3.5-4.4 mm** | 380 | 298 (78.4 [73.9-82.5]) | 6 (1.6 [0.6-3.4]) | 7 (1.8 [0.7-3.8]) | 9 (2.4 [1.1-4.5]) | 44 (11.6 [8.5-15.2]) | 16 (4.2 [2.4-6.8]) |  |
| *2008-2012* | *143* | 115 (80.4 [73.0-86.6]) | <3 (0.7 [0.02-3.8]) | 5 (3.5 [1.1-8.0]) | 5 (3.5 [1.1-8.0]) | 9 (6.3 [2.9-11.6]) | 8 (5.6 [2.5-10.7]) |  |
| *2014-2018* | *207* | 160 (77.3 [71.0-82.8]) | <3 (1.0 [0.1-3.5]) | <3 (1.0 [0.1-3.5]) | 3 (1.4 [0.3-4.2]) | 32 (15.5 [10.8-21.1]) | 8 (3.9 [1.7-7.5]) |  |
| **4.5-5.4 mm** | 236 | 192 (81.4 [75.8-86.1]) | 4 (1.7 [0.5-4.3]) | 10 (4.2 [2.1-7.6]) | <3 (0.8 [0.1-3.0]) | 19 (8.1 [4.9-12.3]) | 9 (3.8 [1.7-7.1]) |  |
| *2008-2012* | *83* | 70 (84.3 [74.7-91.4]) | <3 (0.0 [0.0-4.4]) | 5 (6.0 [2.0-13.5]) | <3 (1.2 [0.03-6.5]) | <3 (2.4 [0.29-8.4]) | 5 (6.0 [2.0-13.5]) |  |
| *2014-2018* | *132* | 104 (78.8 [70.8-85.4]) | 3 (2.3 [0.5-6.5]) | 4 (3.0 [0.8-7.6]) | <3 (0.8 [0.02-4.2]) | 16 (12.1 [7.1-18.9]) | 4 (3.0 [0.8-7.6]) |  |
| **5.5-6.4 mm** | 179 | 155 (86.6 [80.7-91.2]) | <3 (1.1 [0.1-4.0]) | 11 (6.1 [3.1-10.7]) | <3 (1.1 [0.1-4.0]) | 6 (3.4 [1.2-7.2]) | 3 (1.7 [0.4-4.8]) |  |
| *2008-2012* | *74* | 65 (87.8 [78.2-94.3]) | <3 (1.4 [0.03-7.3]) | 4 (5.4 [1.5-13.3]) | <3 (0.0 [0.0-4.9]) | <3 (2.7 [0.3-9.4]) | <3 (2.7 [0.3-9.4]) |  |
| *2014-2018* | *95* | 81 (85.3 [76.5-91.7]) | <3 (0.0 [0.0-3.8]) | 7 (7.4 [3.0-14.6]) | <3 (2.1 [0.3-7.4]) | 4 (4.2 [1.16-10.4]) | <3 (1.1 [0.03-5.7]) |  |
| **≥6.5 mm** | 448 | 272 (60.7 [56.0-65.3]) | 5 (1.1 [0.4-2.6]) | 133 (29.7 [25.5-34.2]) | <3 (0.4 [0.1-1.6]) | 19 (4.2 [2.6-6.5]) | 17 (3.8 [2.2-6.0]) |  |
| *2008-2012* | *184* | 124  (67.4 [60.1-74.1]) | <3 (0.5 [0.01-3.0]) | 46 (25.0 [18.9-31.9]) | <3 (0.5 [0.01-3.0]) | 7 (3.8 [1.5-7.7]) | 5 (2.7 [0.9-6.2]) |  |
| *2014-2018* | *211* | 116 (55.0 [48.0-61.8]) | 3 (1.4 [0.3-4.1]) | 70 (33.2 [26.9-40.0]) | <3 (0.5 [0.01-2.6]) | 11 (5.2 [2.6-9.1]) | 10 (4.7 [2.3-8.5]) |  |
| Data are presented as n (% [95% CI])  SCA: Sex chromosome aneuploidy, CNV: Copy number variation, RAT: Rare autosomal triploidy | | | | | | | | |
